# Supplementary material for: Reactivation of DRP1 plays a functional role in resistance to MEK inhibition in pancreatic cancer cells
Source: bioRxiv. 2026 May 22:2026.05.20.726663. Preprint. [Version 1] doi: 10.64898/2026.05.20.726663 (PMC13228629; doi:10.64898/2026.05.20.726663)
Supplement: 1 [file NIHPP2026.05.20.726663v1-supplement-1.pdf]

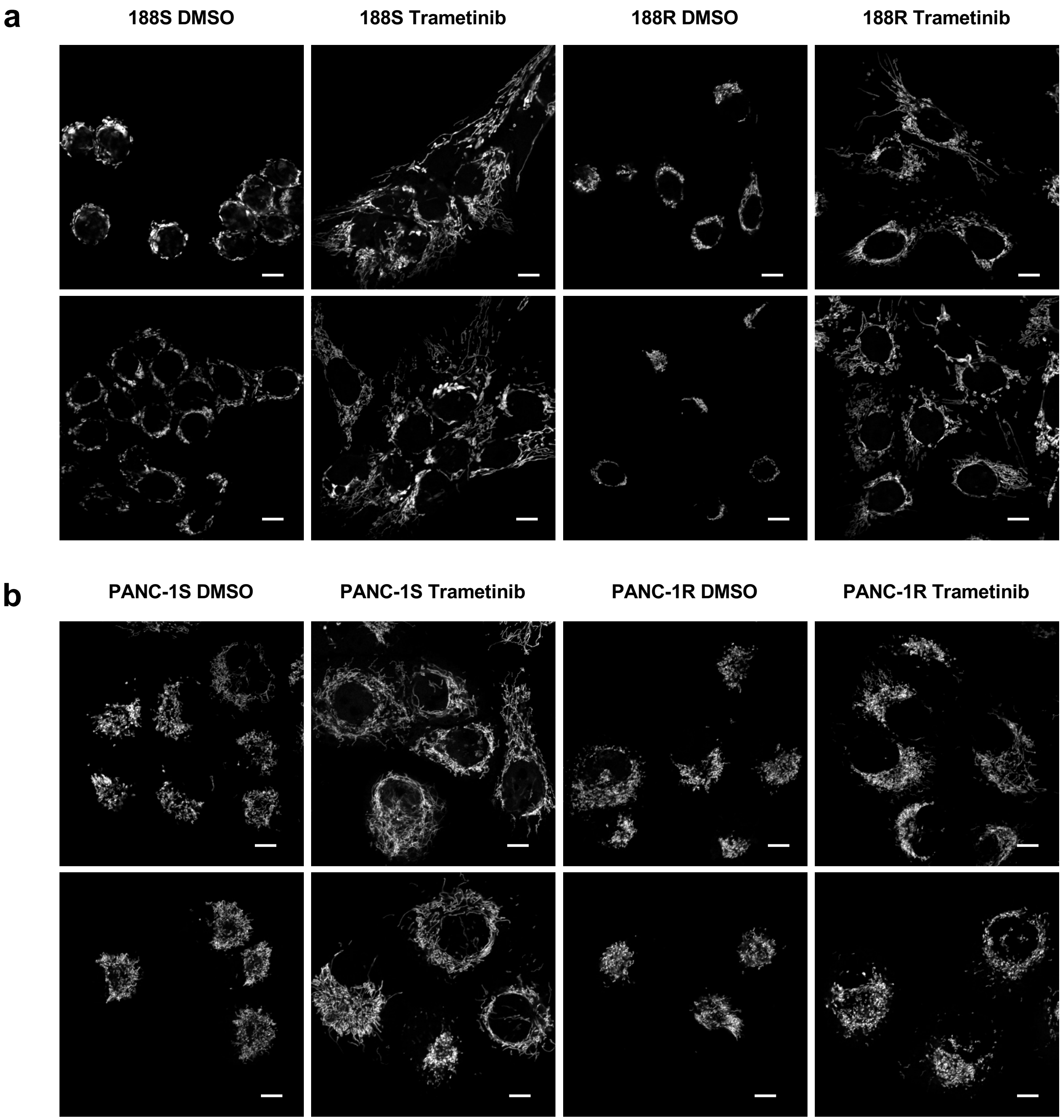

Fig. S1 Trametinib has distinct effects on mitochondrial morphology in trametinib-sensitive and resistant pancreatic cancer cells. 188S and 188R cells (a) or PANC-1S and PANC-1R cells (b) were treated with either DMSO or trametinib (200 nM) for 48hr and stained with MitoTracker red to visualize the mitochondrial network. Representative images from N=3 independent experiments. 30 to 75 cells were analyzed per replicate for each condition.

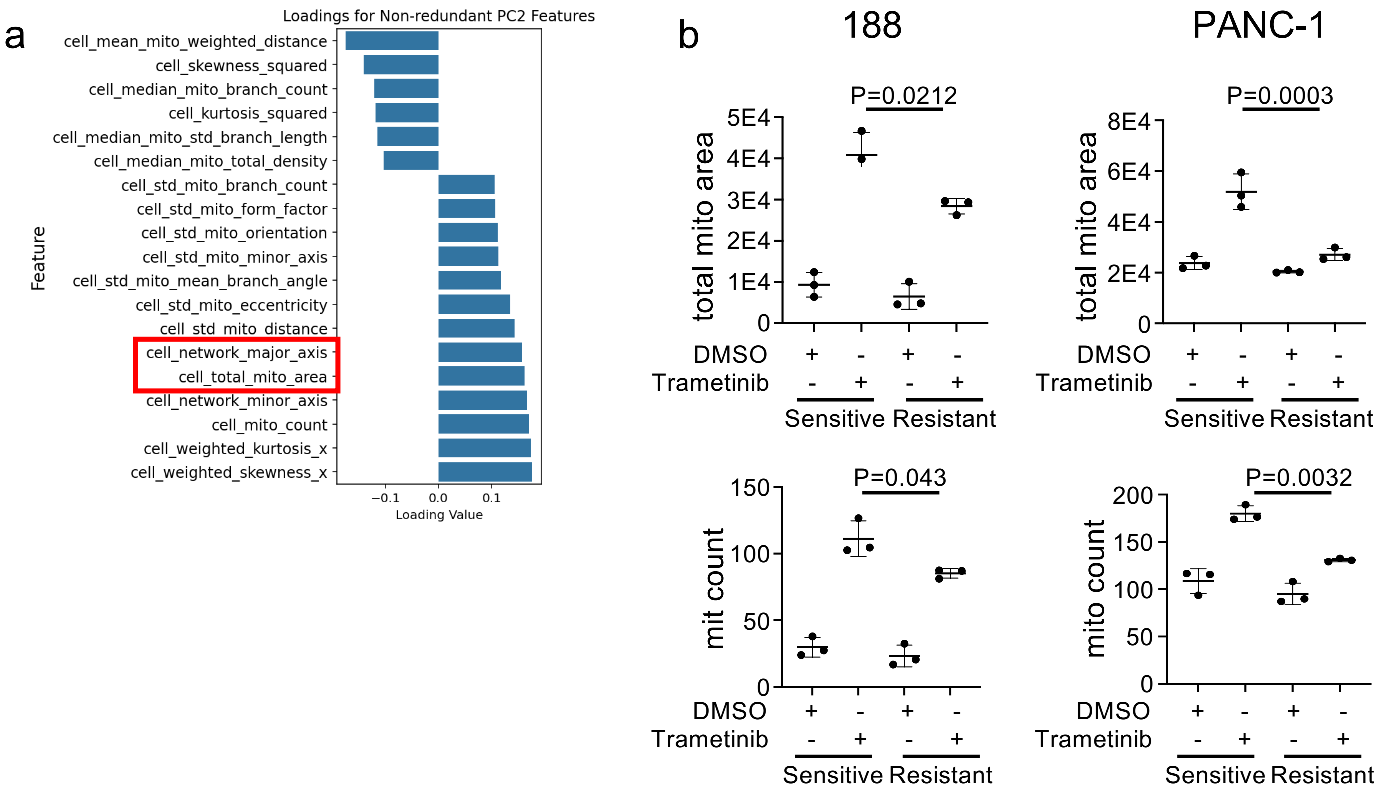

Fig. S2 PCA of mitochondrial features reveals separation between trametinib-resistant and trametinib-sensitive cell lines. PC2 loadings (a) of non-redundant mitochondrial features with the strongest influences on PC2 from the principal component analysis in Figure 3. (b) Representative graphs of differentially regulated features following trametinib treatment. Single-cell data for each mitochondrial feature were averaged to obtain a value for each experimental condition. P values represent two-way ANOVA analysis of sensitive vs resistant cell lines treated with trametinib.

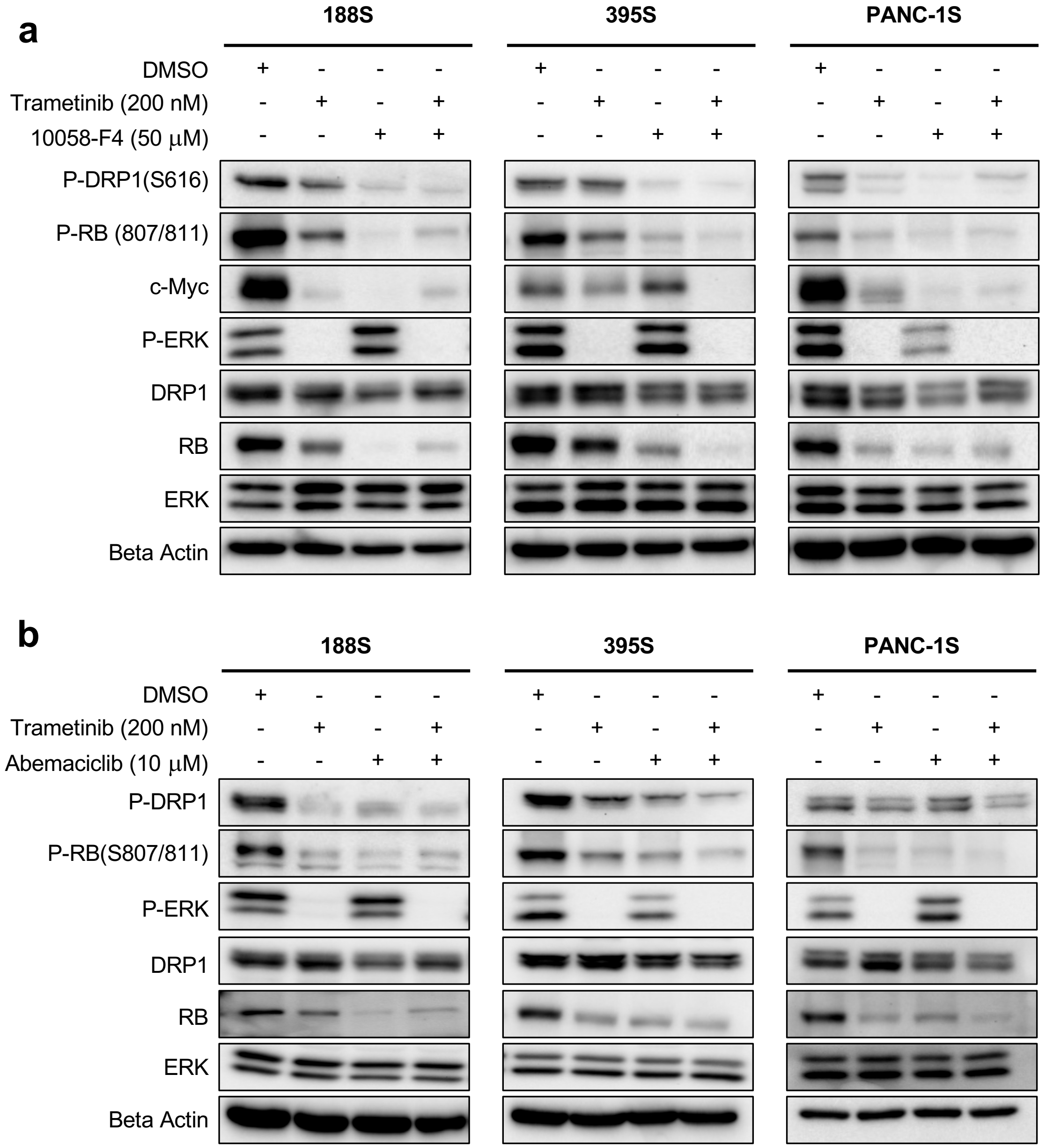

Fig. S3 c-Myc and CDK4/6 contribute to the DRP1 S616 phosphorylation in trametinib-sensitive pancreatic cancer cells. Western blot analysis of the indicated proteins and post-translational modifications from trametinib-resistant 188, 395 and PANC-1 cells following 24hr treatment with: (a) DMSO, trametinib (200 nM), 10058-F4 (50 μM), or a combination of trametinib (200 nM) and 10058-F4 (50 μM); or (b) DMSO, trametinib (200 nM), abemaciclib (10 μM) or combination of trametinib (200 nM) and abemaciclib (10 μM). 20 μg protein was loaded per lane. N=2 independent experiments.

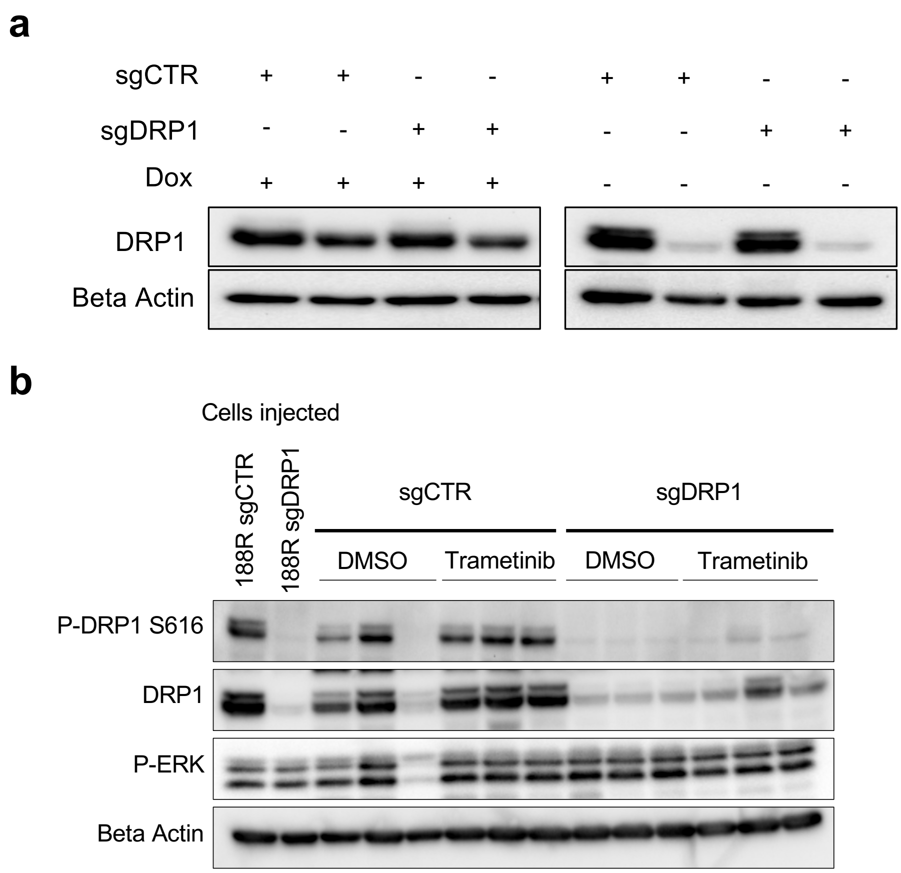

Fig. S4 (a) 188R cells were engineered to express doxycycline-inducible mouse DRP1 followed by either sgCTR or sgDRP1 to knock out endogenous DRP1. Removal of doxycycline leads to loss of mouse DRP1 expression and acute DRP1 knockout. (b) Western blot analysis of lysates generated from the tumors harvested from the experiment shown in Fig. 6g.
